# Isolation and Characterization of Jumbo Coliphage vB_EcoM_Lh1B as a Promising Therapeutic Agent against Chicken Colibacillosis

**DOI:** 10.3390/microorganisms11061524

**Published:** 2023-06-08

**Authors:** Pavel Alexyuk, Andrey Bogoyavlenskiy, Madina Alexyuk, Kuralay Akanova, Yergali Moldakhanov, Vladimir Berezin

**Affiliations:** Laboratory of Antiviral Protection, Department of Virology, Research and Production Center for Microbiology and Virology, Bogenbai Batyr Street 105, Almaty 050010, Kazakhstan; alpagen@live.com (P.A.); anpav_63@mail.ru (A.B.);

**Keywords:** bacteriophage, *Escherichia coli*, colibacillosis, antibiotic resistance, poultry farming

## Abstract

Colibacillosis in chickens can cause the death of young stock, decrease weight gain and lead to significant economic losses. Currently, antibiotic therapy is the main method of treatment of infected animals, but unchecked use of antibiotics has led to widespread antibiotic resistance among microorganisms. Therefore, it is necessary to develop alternative methods of treating bacterial infections that are fully consistent with the One Health concept and introduce them into practice. Phage therapy meets the specified requirements perfectly. This study describes the isolation and characterization of the lytic jumbo phage vB_EcoM_Lh1B and evaluates its potential use in controlling antibiotic-resistant *E. coli* infection in poultry. The complete phage genome is 240,200 bp long. Open reading frame (ORF) prediction shows that the phage genome does not contain genes encoding antibiotic resistance and lysogeny factors. Based on phylogenetic and electron microscopic analysis, vB_EcoM_Lh1B belongs to the group of myoviruses of the *Seoulvirus* genus of the *Caudoviricetes* class. The bacteriophage has good resistance to a wide range of pH and temperatures and has the ability to suppress 19 out of 30 studied pathogenic *E. coli* strains. The biological and lytic properties of the isolated vB_EcoM_Lh1B phage make it a promising target of further study as a therapeutic agent against *E. coli* infections in poultry.

## 1. Introduction

Colibacillosis (escherichiosis) is one of the most common infections in young birds under 80–120 days of age and is the leading cause of mortality and morbidity in poultry farms, resulting in serious economic losses as infected birds continue to die and those that survive are mostly underweight and therefore commercially unviable. Typical treatment strategies include strict control of predisposing factors, administration of antibiotics at the initial stages of the disease and vaccination. Unfortunately, overuse of antibiotics contributes to the formation of antibiotic-resistant *E. coli* serotypes in poultry; besides this, drugs are often used without first determining the sensitivity of microorganisms to them, which undermines the effectiveness of treatment of colibacillosis using this method [1,2]. In addition, according to the One Health concept, to promote human health, it is essential to minimize the use of antibiotics in farm animals, including poultry [3]. In the Republic of Kazakhstan, the national health authorities are taking measures to contain the spread of antibiotic resistance only within the framework of general WHO recommendations. These measures have only slightly reduced the use of antibiotics in medicine. In the meantime, the absence of a national strategy to curb antibiotic resistance in Kazakhstan still creates conditions for mass, uncontrolled and irrational consumption of antibiotics. In addition, the legislation of Kazakhstan lacks any provisions to control the use of antibiotics in agriculture. All this, according to the principles of One Health, leads to a decrease in the effectiveness of the fight against bacterial infections. For example, despite the efforts to control tuberculosis, Kazakhstan ranks 92 out of 138 countries in the prevalence of this disease [4]. Avoiding antibiotics altogether or minimizing their use in general practice will help reduce the spread of antibiotic resistance among pathogens and may even restore antibiotic therapy to its former effectiveness. But in such circumstances, alternatives to antibiotics are needed to combat bacterial infections. Thus, alternative means of growth promotion and control of bacterial pathogens, such as probiotics, prebiotics, enzymes and organic acids, have been suggested to maintain optimal levels of productivity and health in broiler production [5,6,7,8]. Organic acids alone or in combination are generally considered safe and have the same functions as antibiotics [9]. The antimicrobial mechanism of organic acids is thought to lower intestinal pH, thereby limiting the growth of bacteria that are less resistant to acidic pH [10]. Essential oils of plant origin are also attracting increased attention and could be used as a promising alternative to antibiotics due to their effective antimicrobial, antiviral, anticoccidial, antifungal, anti-inflammatory, antioxidant and immunomodulatory effects. In vitro studies have shown that some essential oils, such as thyme, carvacrol, cinnamaldehyde and citral, can inhibit or kill gram-negative and gram-positive bacteria, including *Salmonella*, *E. coli*, *Campylobacter* and *Clostridium perfringens* [11]. Bacteriophages are considered to be one of the most effective substitutes for antibiotics. At the UN General Assembly on 21 September 2016, one of the most popular proposals was to review phage therapy [12]. Bacteriophages are viruses capable of infecting microorganisms. They have inexhaustible diversity, including in shape, size and genome structure [13]. Lytic bacteriophages are promising alternatives for combating antibiotic-resistant forms of human, animal and avian colibacillosis. They have many advantages, such as their ability to lyse bacteria usually highly resistant to antibiotic therapy and form biofilms, as well as their high degree of safety for commensal and symbiotic flora [14]. Phages can be used both in mixtures with other bacteriophage strains, as a phage cocktail, and in combination with various antibiotics, which makes it possible to create complex, multivector, highly effective antibacterial preparations [15,16,17]. There are cases of effective use of various phage preparations for the treatment of colibacillosis in both humans and animals [18,19,20,21]. Phage therapy has proven effective against pathogenic strains of *E. coli*, especially in preventing the development of colibacillosis, which initially develops in the respiratory tract and air sacs and then takes the form of sepsis, causing significant mortality in poultry. Phage suspensions applied directly through the air sac in 3-day-old birds at titres ranging from 10^3^ to 10^6^ PFU/mL for the treatment of *E. coli* infections reduced mortality from 80% in the birds challenged with 10^3^ CFU of *E. coli* to 25 and 5% when mixed with 10^3^ or 10^6^ PFU of the bacteriophage, respectively [22]. In a study by Barrow et al. [20], R bacteriophage was effective in the prevention and treatment of septicemia and cerebritis or meningitis in chickens. Chickens were injected intramuscularly or intracranially with *E. coli*, and phage preparations were administered intramuscularly (in the calf muscle). This application of phage in 3-week-old chickens produced 100% protection against mortality. Other authors compared the efficacy of an antibiotic (chloramphenicol class) and oral phage treatment against enteropathogenic *E. coli* in 20-day-old chickens [21]. In the second week, no diarrhea was detected in birds treated with phages, whereas in birds treated with antibiotics, diarrhea was observed in 12.4% of cases and in the control group (placebo) in 25.2% of cases. Mortality in the control group was 14.8%, which was two and five times higher than in the antibiotic and phage groups, respectively. In addition, phage-treated chickens had higher weight gain. Phage treatment had high specificity without affecting beneficial bacteria, which is very important for maintaining favorable microenvironmental homeostasis in the intestine.

A large number of experimental studies confirm that bacteriophage therapy is very effective against various bacterial infections in poultry; it produces a significant reduction in bacterial infestation or even complete elimination of pathogens. Bacteriophages have been used to protect animals against infections caused by pathogens with significant public health impact, such as *Salmonella enterica*, subsp. enterica serovars Enteritidis, *S. enterica*, subsp. enterica, serovars Typhimurium, *C. jejuni*, *E. coli*, *Listeria monocytogenes* and meticillin-resistant *Staphylococcus aureus* [23,24,25].

Most of the phages isolated and characterized so far possess genomes of fewer than 200 kilobases, but there is a group of phages whose genome exceeds 200 kilobases. This group of viruses is known as jumbo phages, sometimes also referred to as giant phages. For the first time, a phage of this group was isolated from *Bacillus megaterium*—its size exceeded 600 nm [26]. Since then, more than 150 giant phages have been isolated. Over 85% of them have been isolated within the past 4–5 years alone, as bacteriophage research has been rapidly advancing since the development of high-throughput sequencing technology [27]. So far, more than 95% of viruses with large genomes isolated on gram-negative microorganisms belong to the genera *Synechococcus*, *Pseudomonas*, *Caulobacter*, *Vibrio*, *Erwinia*, *Aeromonas* and *E. coli* [28,29]. A relatively small number of isolated phages of this group are associated with the method of concentration of environmental samples using filters capable of cutting off not only microorganisms, but also large phages [30].

In this article, we characterize a new jumbo phage capable of lysing antibiotic-resistant forms of APEC (avian pathogenic *Escherichia coli*). This phage has great potential for use as a therapeutic agent for colibacillosis in poultry, and according to the principles of One Health, this will also contribute to the improvement of human health.

## 2. Materials and Methods

### 2.1. Bacterial Strains

The study used 30 previously archived APEC isolates from post-mortem samples of colibacillosis-suspected chicken collected between 2020 and 2021 from poultry farms of the Almaty region in Kazakhstan. The bacterial isolates were confirmed as APEC using biochemical tests of the Biolog GEN III microorganism identification system (Biolog, Hayward, CA, USA) and real-time PCR for the presence of virulence genes (Supplementary Table S1) [31,32]. The *E. coli* isolates were stored at 4 °C in the collection of the Research and Production Center for Microbiology and Virology.

### 2.2. Determination of Antibiotic Susceptibility of Bacterial Isolates

Antimicrobial susceptibility testing was carried out by rehydrating the wells of the plate with antibiotics using a suspension medium and introducing a bacterial culture into them. The results were interpreted according to EUCAST standards. A MIKROLATEST SENSILAtest kit G-II (Erba Mannheim) was used to determine the antimicrobial susceptibility of bacteria of the *Enterobacteriacea* family. The effect of eight antibiotics belonging to different groups (monobactams, third- and fourth-generation cephalosporins, carbapenems, aminoglycosides, ureidopenicillins and glycylcyclines) on the growth of bacterial isolates was investigated. Antimicrobial susceptibility testing of bacterial cultures was performed twice.

### 2.3. Bacteriophage Isolation

The bacteriophage was isolated from coastal soil samples collected at the mouth of the Ural River (Northern coast of the Caspian Sea). The culture of *E. coli* ATCC 25922 was used for bacteriophage isolation. Some 5 g of the soil sample was poured with 50 mL of sterile SM buffer (50 mmol/L Tris-HCl, pH: 7.5; 0.1 mol/L NaCl; 8 mmol/L MgSO_4_), and then the sample was homogenized and incubated on an orbital shaker at 100 rpm for 6 h. The obtained homogenate was centrifuged at 4000× *g* for 10 min, and the supernatant was filtered through 0.45 µm bacterial filters (Nalgene Syringe Filter; Thermo Scientific, Waltham, MA, USA). The resulting filtrates were stored in sterile conditions at 4–8 °C.

For bacteriophage isolation, 1 mL of 10× nutrient broth (Condalab, Madrid, Spain), 1 mL of indicator bacteria suspension with a turbidity of 0.5 McFarland units (MFU) (10^8^ CFU/mL) and 100 µL of MgSO_4_ were added to 10 mL of the prepared filtrate. The mixture was incubated at 37 °C for 18 h. At the end of the incubation, the mixture was centrifuged at 4000× *g* for 15 min and then filtered through 0.45 µm bacterial filters (Nalgene Syringe Filter; Thermo Scientific) to remove any bacterial cells.

### 2.4. Determination of Bacteriophage Titer

The presence of a bacteriophage was confirmed and its titer determined by the method of inoculation with a bacterial culture on double-layer agar (DLA or Gratia method) [33]. Ten-fold serial dilutions (10–10^−9^) of the above filtered phage suspensions were prepared using a sterile phosphate buffer (pH 7.2, AMRESCO^®^, Solon, OH, USA). Equal volumes (1 mL) of the diluted phage and host *E. coli* with a turbidity of 0.5 MFU containing 10^8^ CFU/mL were mixed with 2.5 mL of 0.7% nutrient agar (Condalab, Madrid, Spain). The prepared mixture was poured into Petri dishes onto the surface of a previously prepared and solidified 2% nutrient agar and incubated for 18 h at 37 °C. The same mixture was used as a control but with 1 mL of a sterile phosphate buffer instead of the aqueous sample. At the end of the incubation period, plaque-forming units (PFUs) were observed.

### 2.5. Bacteriophage Purification and Propagation

After PFUs were formed on the double-layer agar, a number of clear plaques of the same morphotype (size and shape) were selected for further purification by successive single plaque isolation from the higher dilution plates where the plaques were distinct. A clear lysis zone of a specific morphotype was selected at the maximum dilution of the phage lysate, picked up from the agar and placed in 5 mL of sterile nutrient broth, incubated on an orbital shaker at 100 rpm for 2 h at 37 °C, after which the procedure of enrichment with bacteriophages was carried out, and again titrated on double-layer agar. This procedure was repeated at least three times until PFUs of only one morphotype were obtained [34,35].

Some 40 mL of sterile nutrient broth was poured into sterile 50 mL test tubes, and then 500 µL of propagated bacteriophage and 5 mL of a 24 h culture of host bacteria were added and cultivated on an orbital shaker at 100 rpm for 24 h at 37 °C. After the cultivation, the resulting phage lysates were successively purified by centrifugation for 30 min at 4000× *g* and filtration through 0.45 μm membrane filters. The phage lysates were stored in sterile conditions at a temperature of 4–8 °C.

### 2.6. Multiplicity of Infection (MOI)

To determine the multiplicity of infection (MOI) of the bacteriophage, a bacterial suspension at a concentration of 10^8^ CFU/mL was mixed with a bacteriophage suspension in various ratios (0.0001, 0.001, 0.01, 0.1, 1 and 10 PFU/CFU). After incubation at 37 °C for 4 h, the samples were centrifuged and filtered to remove bacteria [36]. The filtrates were titrated by the Gratia method to determine the bacteriophage titer [33]. A bacteria-free suspension and a bacteriophage-free suspension were included in all the experiments as controls. The ratio of MOI with the highest bacteriophage titer at the output was considered optimal. This experiment to determine the multiplicity of infection was performed at least in triplicate.

### 2.7. Host Range Determination

Host ranges were determined by co-cultivation of isolated bacteriophages with various *E. coli* strains in microplates. Some 150 μL of nutrient broth, 50 μL of the phage-containing sample (10^8^ PFU/mL) and 50 μL of a bacterial suspension (10^8^ CFU/mL 0.5 MFU) were put into the wells of the microplate. Samples containing 50 µL of sterile nutrient broth instead of bacteriophages were used as a control for the growth of the bacterial test culture. As a negative control, a sample containing only 200 µL of sterile nutrient broth and 50 µL of the original phage lysate was used. Cultivation was carried out at 37 °C in an Infinite^®^ 200 PRO plate reader (Tecan, Männedorf, Switzerland). The dynamics of bacterial growth was assessed by the change in the optical density of the suspension at 600 nm. Microplates were scanned every 30 min during the entire cultivation period [37]. Each experiment was repeated three times. To set up benchmarks, the optical density corresponding to 100% inhibition of bacterial growth was taken as that of the samples without any bacterial suspension added (negative control), and the optical density corresponding to 0% inhibition of bacterial growth was taken as that of the samples without any phage lysates added (positive control).

### 2.8. Influence of Temperature and pH on Bacteriophage Stability

Tests for phage resistance to external factors were executed following the protocols described previously with some modifications. The titer of the bacteriophage was determined by the Gratia method [38].

For temperature stability analysis, the vB_EcoM_Lh1B phage suspension was diluted in SM buffer (1:9 dilution) and incubated for 1 h at various temperatures (15, RT, 37, 40, 50 and 90 °C). A bacteriophage suspension kept at 4 °C in the SM buffer was used as a control in this study. After incubation, the titer of the bacteriophage was determined by the double agar overlay plaque assay.

For pH stability analysis, 100 µL of the vB_EcoM_Lh1B phage suspension was diluted in 900 µL of SM buffer with various pH values (2.0, 4.0, 7.0, 10.0 and 12.0) prepared using 1 M HCl or 1 M NaOH. The tubes were incubated for 1 h at 37 °C. Next, 10-fold serial dilutions of the contents of each tube were prepared in SM buffer and used for plating. After an overnight incubation at 37 °C, the PFUs were counted. The number of phage plaques incubated in SM buffer with a pH value of 7.0 was used as a control in this study. Experiments on the effect of temperature and pH on the bacteriophage’s stability were performed twice.

### 2.9. Adsorption Assay and One-Step Growth Curve

Adsorption and one-step growth assays were performed as previously described [39,40]. The host bacterial strain at 10^8^ CFU/mL (0.5 MFU) was co-cultured with the bacteriophage at a titer of 10^8^ PFU/mL (MOI 1) at 37 °C. For adsorption analysis, 100 μL samples were taken at 0, 3, 5, 10 and 20 min. Each sample was centrifuged to precipitate bacterial cells, and the supernatant was used to determine the amount of unadsorbed phages.

For one-step growth assay, the bacteriophage suspension was mixed with 1 mL of host bacterial culture at an MOI ratio of 0.1. The mixture was incubated at 37 °C and 150 rpm for 5 min followed by centrifugation at 10,000× *g* for 10 min. The pellet was resuspended in 10 mL TSB and incubated at 37 °C and 150 rpm. Some 100 µL of samples were taken at 10 min intervals for a total of 1 h and enumerated by double agar overlay plaque assay in duplicate. The bacteriophage latent period and burst size were estimated using a growth curve [41]. All experiments were carried out at least in triplicate.

### 2.10. Concentration of Bacteriophages by Ultracentrifugation

The bacteriophages were sedimented from the phage lysate by centrifugation at 100,000× *g* for 90 min at 4 °C in an Avanti J-30i centrifuge (Beckman Coulter, Brea, CA, USA). The resulting bacteriophage sediment was dissolved in a minimum volume of PBS [42].

### 2.11. Electron Microscopy

For transmission electron microscopy, 30 microliters of concentrated bacteriophage was spotted on a formvar-coated copper grid (300 mesh) and incubated for 1 min. Then, 3% phosphotungstic acid (pH 6.8) was used to stain the samples, and excess solution was removed. The grids were allowed to air dry and were then observed with a JEOL JEM-2100 (JEOL, Ltd., Akishima, Tokyo, Japan) transmission electron microscope (voltage 100 kV). At least 20 phage images were used to assess the particle dimensions [43].

### 2.12. Phage DNA Isolation and Sequencing

The genomic DNA from the phage lysate was extracted according to the guide for the PureLink viral DNA/RNA minikit (Thermo Fisher Scientific, Waltham, MA, USA). The extracted DNA was diluted to 0.2 ng/µL and prepared for whole-genome sequencing. Libraries were prepared using the Nextera XT Kit (Illumina, Cambridge, UK) according to the manufacturer’s instructions. Library concentrations were determined using the Qubit 3.0 and Qubit dsDNA High Sensitivity Kit. Libraries were sequenced on the Illumina MiSeq platform using MiSeq Reagent Kits v3 (2 × 300 bp).

### 2.13. Bioinformatic Analysis

After sequencing, low-quality reads were filtered out and adapters trimmed using Trimmomatic from the Genome Detective tool [44,45]. The average read length after trimming was 286 bp. Genomes were assembled from paired reads using SPAdes 3.12.0 (an installable plugin in Geneious Prime) [46]. The genomes were assembled to an average depth of more than 300. The genomic sequence of *E. coli* phage vB_EcoM_Lh1B was annotated by predicting and validating open reading frames (ORFs) using the ORF finder tool accessed on 1 August 2022 (https://www.ncbi.nlm.nih.gov/orffinder/, accessed on 1 August 2022) and Geneious Prime using methionine and alternative initiation codons as start codons. The putative coding sequences (CDSs) were predicted using BLASTp of the predicted ORFs against the NCBI non-redundant protein sequences (nr) database. To increase the confidence in the predicted ORFs and CDSs, they were compared to those predicted through PHASTER [47]. tRNA coding regions were identified with tRNAscan-SE [48].

Sequences of phages from different genera of the *Caudoviricites* class were imported into Geneious Prime to draw a phylogenetic tree using the following default parameters: CLUSTAL-W aligner and FastTree plugin using the maximum likelihood method with 1000 bootstrap replications. Phylogenetic analyses were performed using the major capsid protein. The comparative genome analysis of whole genomes of some jumbo viruses was performed using Mauve [49]. Genetic maps targeting putative coding genes were generated using SNAPGene.

### 2.14. Statistics and Graphic Display of Results

Statistical analysis of the results was carried out using Statistica 10.0 and Microsoft Excel. Microsoft Office was then used to create tabular and graphic representations of the findings. A one-way ANOVA test was conducted to indicate any significant difference of bacterial concentration between control and bacteriophage treatment. Values for all of the parameters were expressed as the mean ± the standard deviation (SD). *p*-values less than 0.05 were considered statistically significant.

## 3. Results

### 3.1. Determination of the Antibiotic Resistance of Isolates

**Figure 1:**
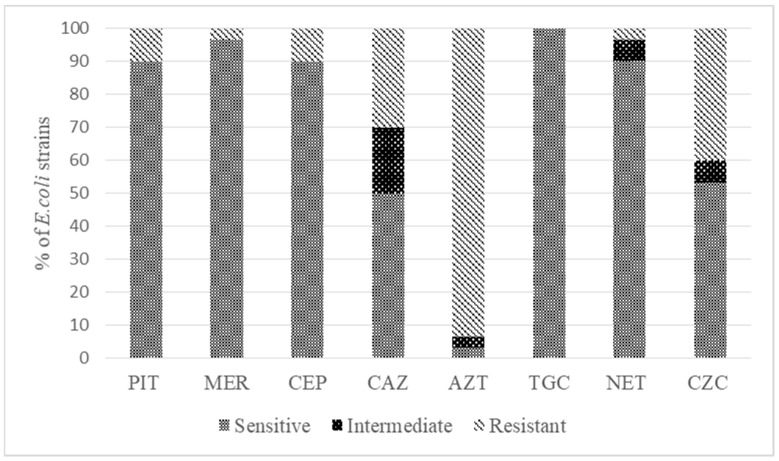
Antimicrobial susceptibility test results for *E. coli* isolates. The bars represent the percentages of the 30 *E. coli* isolates that were resistant, intermediate or susceptible to the eight antibiotics. PIT—piperacillin/tazobactam, MER—meropenem, CEP—cefepime, CAZ—ceftazidime, AZT—aztreonam, TGC—tigecycline, NET—netilmicin and CZC—ceftazidime/clavulanate.

As a result of the studies, it was found that 93% of the *E. coli* isolates were resistant to aztreonam, 40% to ceftazidime/clavulanate, 30% to ceftazidime and 10% to piperacillin-tazobactam and cefepime. The smallest number of isolates (3.3%) were resistant to meropenem and to netilmicin. In addition, none of the studied isolates showed resistance to tigecycline (Figure 1, Supplementary Table S2). Thus, it was found that 10 of the 30 studied *E. coli* strains were resistant to two or more groups of antimicrobials (MDR = 33.3%).

### 3.2. Bacteriophage Isolation and Plaque Morphology

The bacteriophage was isolated from coastal soil samples collected at the mouth of the Ural River (Northern coast of the Caspian Sea). Clear plaques were formed by bacteriophages on the lawn of *E. coli* ATCC 25922 using the double agar overlay method. Using the method of successive selective passages, a pure phage line named vB_EcoM_Lh1B was obtained.

**Figure 2:**
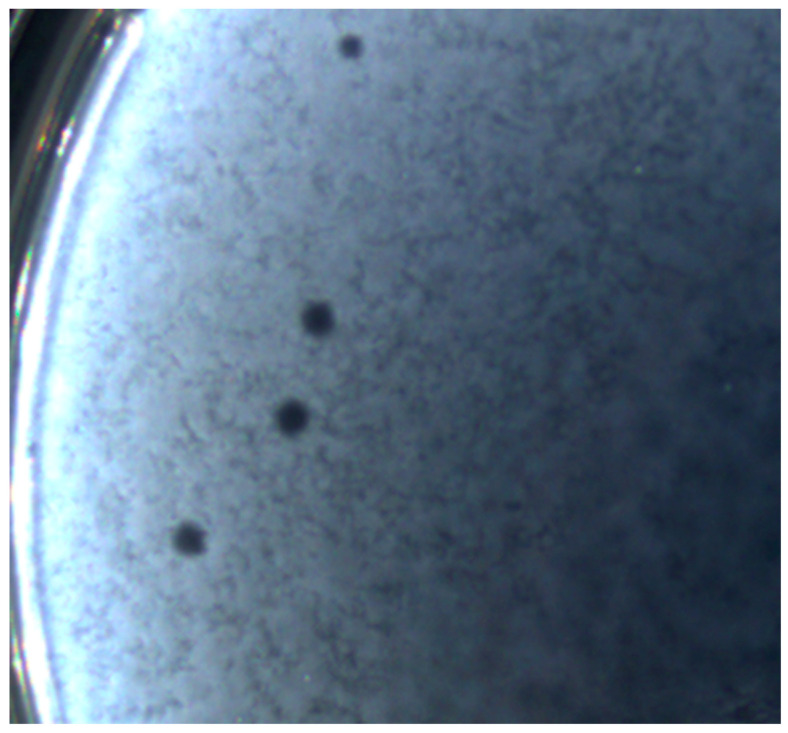
Plaques formed by the vB_EcoM_Lh1B bacteriophage on the lawn of *E. coli* ATCC 25922 using the double agar overlay method; incubation at 37 °C for 18 h; 5× magnification.

Further analysis revealed that the studied bacteriophage formed clear plaques on the host’s lawn and produced complete lysis in moderate titers (5.0–9.0 × 10^8^ PFU/mL). The vB_EcoM_Lh1B plaques shown in Figure 2 had an average diameter of about 3.5 mm. The clear zone of lysis without culture regrowth indicated that the virus was a lytic bacteriophage.

### 3.3. Determination of Multiplicity of Infection, Adsorption Rate and One-Step Growth Curve of the Bacteriophage

**Figure 3:**
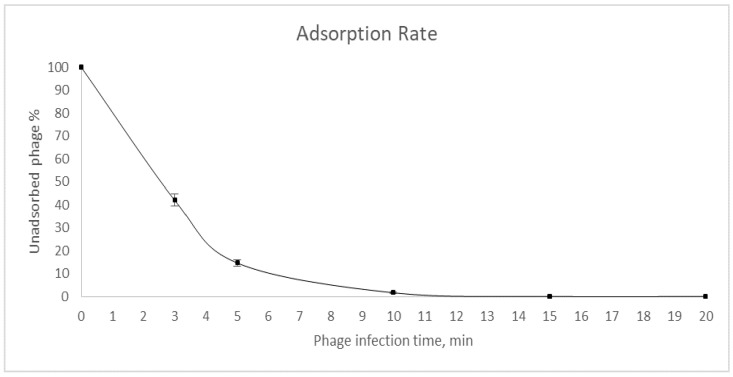
Adsorption rate of the vB_EcoM_Lh1B phage.

The multiplicity of infection assay showed that the vB_EcoM_Lh1B bacteriophage produced the highest yield of viral particles at MOI values of 0.1 to 1 (1.5 × 10^9^ PFU/mL to 2.5 × 10^9^ PFU/mL, respectively). The study of virus adsorption showed that after just 5 min, about 80% of the virus was adsorbed to the host cell *E. coli* ATCC 25922 (Figure 3).

**Figure 4:**
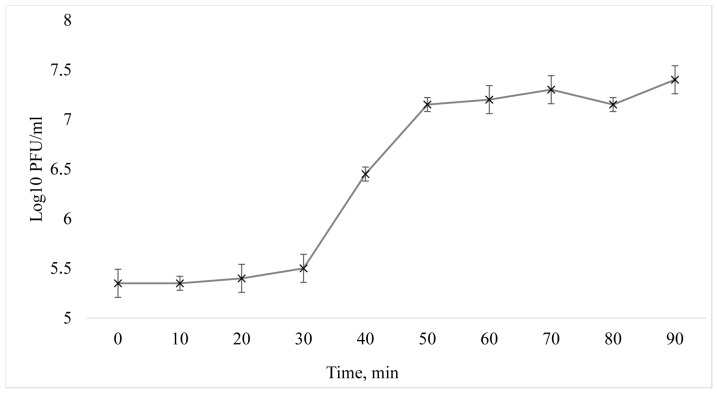
One-step growth curve of the vB_EcoM_Lh1B phage.

From the one-step growth curve, the vB_EcoM_Lh1B bacteriophage is seen to have a latent period of 30 min followed by a burst size period of up to 50 min, followed by a growth plateau (Figure 4). The average burst size was 134 plaque-forming units (PFU) per cell.

### 3.4. Bacteriophage Morphology

**Figure 5:**
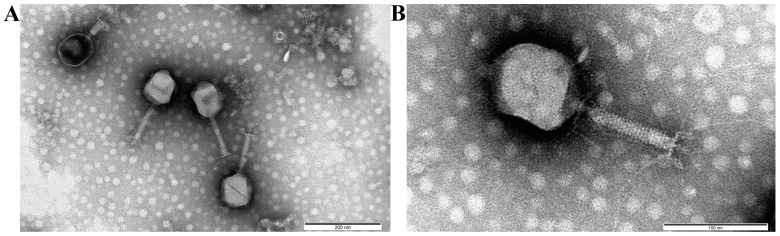
Transmission electron micrographs of the vB_EcoM_Lh1B bacteriophage. (**A**) 50,000 magnification (scale bar = 200 nm); (**B**) 150,000 magnification (scale bar = 100 nm).

A study of the vB_EcoM_Lh1B phage using transmission electron microscopy showed that the size of the virion corresponded to the size of giant phages and averaged 198 ± 7 nm; the tail length averaged 98 ± 7 nm, and the head diameter was 75 ± 11 nm. At the same time, the electron micrograph clearly revealed that the studied virus belonged to the myoviruses group as its icosahedral head, tail surrounded by a contractile sheath and the characteristic base plate with long tail fibers are clearly visible in the images (Figure 5).

### 3.5. External Factors Stability Tests on the Phage

**Figure 6:**
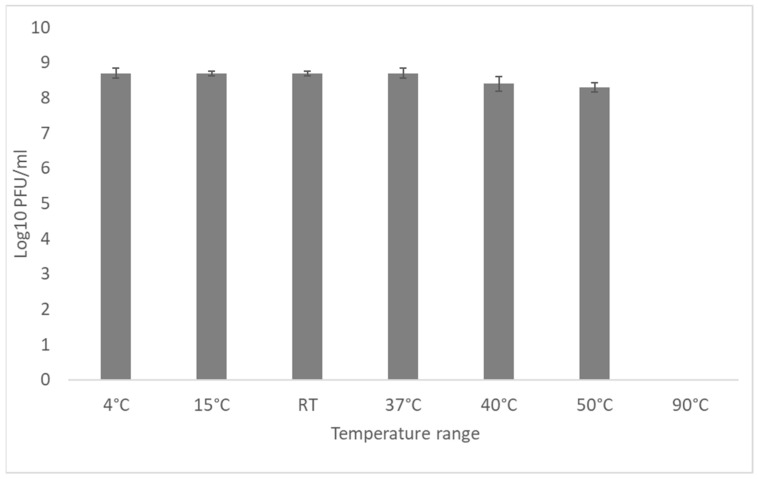
Stability of the vB_EcoM_Lh1B bacteriophage at different temperatures. These values represent the average of duplicate tests.

The thermostability of the vB_EcoM_Lh1B bacteriophage was evaluated by incubating it for 60 min at a range of temperatures from 4 to 90 °C. It showed that the studied bacteriophage retained its lytic activity at the level of 10^8^ PFU/mL at temperatures of 4, 15, RT, 37, 40 and 50 °C. When incubated at 90 °C, the phage titer dropped sharply below the detection level (Figure 6). These results show that the investigated bacteriophage is able to tolerate standard ambient temperatures.

**Figure 7:**
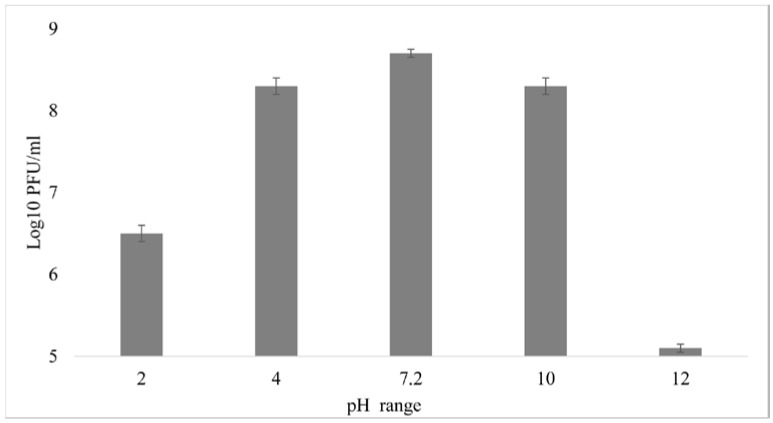
Stability of the vB_EcoM_Lh1B bacteriophage in different pHs. These values represent the average of duplicate tests.

A study of the vB_EcoM_Lh1B bacteriophage stability in different pHs showed that its exposure to pH 4.0–10.0 for 60 min had practically no effect on the final titer of viral particles, which in all the cases averaged 10^8^ PFU/mL, while at pH 2 the titer decreased to 10^6^, and at pH 12 to 10^5^ (Figure 7). It was found that the studied bacteriophage is highly stable at standard values of acidity and temperature. Extreme temperatures and pH of the medium either completely inactivated the studied bacteriophage or reduced the titer of active viral particles as much as 1000 times (Figure 6 and Figure 7).

### 3.6. Host Range Determination

The activity of bacteriophage vB_EcoM_Lh1B was determined against 30 strains of avian pathogenic *E. coli* strains and was evaluated based on changes in the growth dynamics of bacterial cultures during co-cultivation for 8 h. The MOI value was 1 in all cases. The dynamics of bacterial growth were determined by the change in the optical density of the culture medium at 600 nm. As a result, it was found that 19 out of 30 tested *E. coli* strains showed sensitivity to the vB_EcoM_Lh1B phage: the studied bacteriophage completely inhibited the growth of four bacterial cultures, suppressed the growth of seven cultures by 50–90% and partly suppressed the growth of the remaining eight bacterial cultures, with the effect ranging from 25% to 44% (Table 1).

### 3.7. Bioinformatic Analysis of the vB_EcoM_Lh1B Bacteriophage Genome

The sequence of the complete genome of the vB_EcoM_Lh1B phage was obtained by sequencing on the Illumina platform (Shotgun sequencing) with subsequent assembly in the Geneious Prime (version 2022.2.2) software package [50].

**Figure 8:**
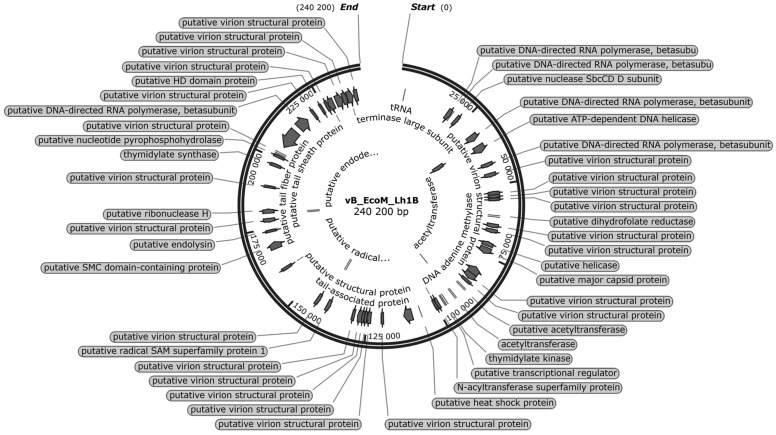
Genome map of the vB_EcoM_Lh1B phage. Only functional genes are indicated.

ORF prediction using the standard genetic code identified 270 putative protein-coding genes, in addition to the tryptophan-tRNA gene. This phage had 236 open reading frames on the leading strand and 34 ORFs on the complementary strand. In addition, 213 genes encoding hypothetical proteins and 57 functional genes were identified in the vB_EcoM_Lh1B phage genome, of which 44 ORFs were located on the leading strand and 13 ORFs on the complementary strand. The content of GC pairs was 48.5%. Virion structural proteins, DNA packaging and DNA replication/transcription/repair proteins were identified among functional phage proteins (Figure 8). Genes encoding factors for lysogeny and antibiotic resistance were not found.

**Figure 9:**
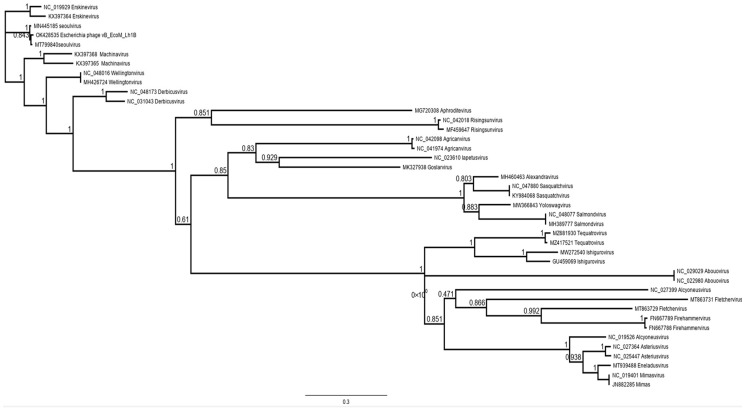
Phylogenetic analysis of major capsid protein sequences of representatives of several genera of the *Caudoviricites* class.

To further investigate the phylogenetic relationship of jumbo phage vB_EcoM_Lh1B, genome sequences of viruses of several genera of the *Caudoviricetes* class were evaluated. Phylogenetic analyses were performed using the major capsid protein. The results showed that the isolated phage formed a cluster with viruses of the *Seoulvirus* genus (Figure 9).

**Figure 10:**
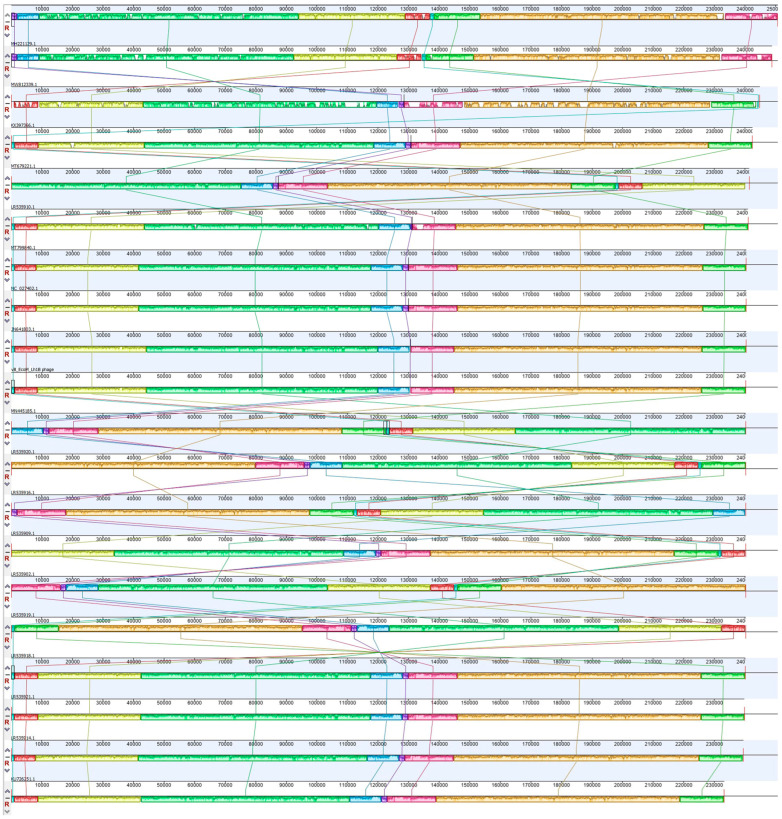
Mauve alignment of a group of jumbo viruses and bacteriophage vB_EcoM_Lh1B.

A whole-genome comparison of bacteriophage vB_EcoM_Lh1B with other members of the jumbo phage group using the Mauve alignment method showed that the studied virus is similar in its genome structure to the following viruses: Salmonella phage JN03 (MT799840.1), Salmonella phage SPN3US (NC_027402.1), Salmonella phage SPN3US (JN641803) and Escherichia phage vB_EcoM_EC001 (MN445185) (Figure 10).

## 4. Discussion

According to the One Health concept, human health largely depends on the health of the environment and the quality of consumed food and water. The validity of this concept is further emphasized by the current problem of the global spread of antibiotic resistance among microorganisms. Thus, the unchecked and unjustified use of antimicrobial drugs in animal husbandry and crop farming poses serious threats to human life and health [51,52,53]. Many antimicrobials such as ampicillin, gentamicin and erythromycin used in livestock are similar or closely related to those used to treat humans. Consequently, since 2005, the World Health Organization (WHO) has compiled a list of Critically Important Antimicrobials (CIA) requiring surveillance to reduce the spread of antimicrobial resistance and preserve drugs that are important to human medicine, which includes antibiotics that should not be used in veterinary medicine except under certain conditions. [54]. Frequent usage of antibiotics in commercial poultry farming for growth promotion and disease prevention is seen as one of the major risk factors or drivers for the emergence of antibiotic-resistant bacteria [55,56]. This increases the risk of transmission of antibiotic resistance properties to bacterial pathogens, hence significantly reducing the effectiveness of antibiotic therapy. In treatment of bacterial infections, this can lead to the development of serious complications and even cause death. In addition, the emergence of multidrug-resistant (MDR) bacteria poses increasing challenges to veterinarians in providing effective treatment for farm animals [57,58]. Several studies have reported resistance of *E. coli* isolates to some drugs such as tetracycline, ampicillin, sulphamethoxazole/trimethoprim, ciprofloxacin, gentamycin and nalidixic acid [59]. All of the above has stimulated the renewed interest of scientists in bacteriophages since the beginning of the 21st century. Bacteriophages are viruses of bacteria. They are ubiquitous in nature and accompany bacteria in every environment they colonize, including the human and animal microbiota [23].

In this study, a new bacteriophage vB_EcoM_Lh1B was isolated; it has lytic properties in relation to pathogenic strains of *E. coli* isolated from birds. The isolated bacteriophage was classified as a member of the myovirus group based on the results of transmission electron microscopy. On the electron micrograph, the bacteriophage had an elongated hexagonal head and a tail surrounded by a contractile sheath with a well-defined basal plate with long tail filaments. The results of whole-genome sequencing made it possible to identify bacteriophage vB_EcoM_Lh1B as a representative of the *Seoulvirus* genus of the *Caudoviricetes* class, which confirms the findings of the electron microscopy.

The stability of a phage under various environmental conditions is an important characteristic for both survival and selection of phages for therapy. Various studies have shown that different phages exhibit different levels of resistance to physical and chemical factors [17,60,61]. In this study, various pHs and temperature ranges were chosen to approximately mimic the conditions that may occur when phages are used as a therapeutic means or disinfectant in poultry farms. The obtained results showed that the vB_EcoM_Lh1B phage titer remains practically unchanged at 10^8^ PFU/mL in temperatures ranging from 4 to 50 °C, which is consistent with other studies [62].

Sixty minutes of incubation of the vB_EcoM_Lh1B phage at 90 °C resulted in its complete inactivation, which is normal for bacteriophages and fully consistent with previous studies by Lu et al., Yu, Y.P. et al. and Shende et al., who reported that phages are inactivated at temperatures of 70 °C or higher [63,64,65]. The findings suggest that the investigated bacteriophage can be stored at room temperature, which is especially important amid limited resources. In addition, the body temperature of warm-blooded animals (birds and mammals) will not lead to inactivation or decrease of the lytic activity of the vB_EcoM_Lh1B phage upon its introduction.

A study of pH stability revealed that the phage remained viable in the pH range of 4.0–10.0. Moreover, even at more extreme levels of pH 2.0 and pH 12.0, the phage titer did not drop below the detection limit, but remained at 10^6^ and 10^5^ PFU/mL, respectively, which also corresponds to the data obtained during the study of other coliphages [62].

However, some studies have shown that the studied phages retain their initial titer of viral particles only at pH 7.0–8.0 [66] and that the virions of the studied bacteriophages do not withstand extreme pH levels (2.0 and 12.0) [67]. Thus, in studies of the biological properties of lytic myophage MJ1 against multiresistant strains of E.coli, it was found that this bacteriophage had maximum stability at pH 7 and was also relatively stable at pH 5 and 9. Extreme pH values below 3 and above 12 were lethal and completely inactivated the phage [68]. The vB_EcoM_Lh1B bacteriophage showed good stability at the pH range of 2–12, which simplifies its storage, and suggests that the acidic environment of the stomach will not lead to its inactivation upon oral administration to animals and humans.

According to studies conducted by W.E. Huff et al. into the effect of bacteriophage titer on the treatment of colibacillosis in broiler chickens, the therapeutic efficacy of bacteriophage is little dependent on the MOI and it should not be used as a basis for treatment of natural diseases with bacteriophages [69]. However, the success of phage infection depends on a chance meeting with the target bacterium for its infection and lysis. If bacteriophages reach the site of a bacterial infection, they are effective in eliminating the infection. Therefore, in the case when the bacterial infection is generalized and affects the whole organism, an increase in the MOI may be appropriate in order to increase the number of cases of phage encounters with target bacteria and thereby increase the effectiveness of phage therapy. In vitro and in vivo experiments to study the effectiveness of phage against bacteria traditionally use MOI ranging from 0.01 to 100. Most often, the MOI is 100 in order to guarantee the presence of a sufficient number of phages in the medium. However, not all phages multiply or survive equally. It is important to determine the lytic replication cycles and the phages’ resistance to environmental conditions [70]. In our study, it was determined that the optimal MOI value for the propagation of the vB_EcoM_Lh1B bacteriophage is in the range from 0.1 to 1. At these MOI values, the highest yield of viral particles was observed.

Myoviruses are known to have a shorter life cycle, so the previously characterized myophage (phage Enterobacter myPSH1140) had a latent period of 11 min and a burst size of 135 phages per infected cell [71]. It was noted that the number of daughter phage particles (burst size) significantly depends on the presence of bacterial host cells [72]. Therefore, bacteriophage life-cycle parameters play an important role in determining phage activity both in vitro and in vivo (during therapy), since phage multiplication is directly proportional to the decrease in the number of bacteria. In our studies, the vB_EcoM_Lh1B bacteriophage demonstrated a high adsorption rate on bacterial cells. The bacteriophage one-step growth curve showed that the latent period was approximately 30 min, which is a relatively short period compared to other *E. coli* phages [66]. Therefore, the observed relatively short latency period of the vB_EcoM_Lh1B phage is a desirable characteristic for its potential application in phage therapy.

Studies performed to determine the host range of the vB_EcoM_Lh1B phage showed that it is able to suppress the growth of 19 out of 30 tested cultures. Such a wide range of hosts of this virus can be explained by its belonging to the group of myoviruses: ecological studies have shown that myophages have a wider range of hosts than siphophages and podophages [73], possibly due to the structural features of the contractile tail, in particular the structure of the basal plate [74].

Whole-genome sequencing of the vB_EcoM_Lh1B phage and nucleotide sequence assembly established a genome length of 240,200 bp, indicating that the bacteriophage belongs to jumbo phages of the *Caudoviricetes* class. The phylogenetic tree constructed using FastTree based on the sequence of the major capsid protein showed that its closest relative is the Escherichia vB_EcoM_EC001 phage (GenBank accession number MN445185) and that it is affiliated with the *Seoulvirus* genus.

Moreover, whole-genome sequence analysis and functional annotation showed that the vB_EcoM_Lh1B phage did not encode genes of lysogeny factors (genes of transposases, integrases, prophages, etc.) and antibiotic resistance. Despite conflicting opinions regarding the use of bacteriophages in veterinary medicine, and in poultry in particular, our study confirms the possibility of using bacteriophages as an effective means of combating colibacillosis in chickens.

## 5. Conclusions

Our study have shown that resistance to a wide range of temperatures and pH, as well as a broad range of hosts of the isolated lytic phage vB_EcoM_Lh1B make it a potential therapeutic agent against *E. coli* infections in poultry.

## Figures and Tables

**Table 1:** *E. coli* strains and their susceptibility to the phage.

APEC Isolate ID	The Level of Lytic Activity of the vB_EcoM_Lh1B Phage *	APEC Isolate ID	The Level of Lytic Activity of the vB_EcoM_Lh1B Phage
*E. coli* F/18	100% +++	Int WP #4	30% +
Sor. lv. bc2	100% +++	Br. m/L 10.06	28% +
Yun 3d pas	100% +++	Sor. st. Sc1	25% +
Sor. lb. sc2	100% +++	Alag 1 st.	25% +
Int. m/L 04.06	85% ++	Sor. st. bc1	-
Sor. br. sc2	75% ++	Int WR #4	-
Sor. int. bc2	64% ++	Yun#2	-
Kd. m/L 04.06	61% ++	St. m/L 04.06	-
Sor. st. sc2	58% ++	Lv. m/L 04.06	-
Faec. H.bl. Uz.	55% ++	St. m/L 10.06	-
Sor. br.bc1	51% ++	F.ch. m/L Uz.	-
Sor. kd. sc2	44% +	Sor. lv. Bc1	-
Sor. lv. sc2	43% +	Sor. lv. Sc1	-
Sor. br. sc1	42% +	Sor. kd. bc1	-
F.H. pr. Uz.	34% +	Sor. br.bc2	-

* +++—Inhibition of bacterial culture growth by 100–90%; ++—inhibition of bacterial culture growth by 90–50%; +—inhibition of bacterial culture growth by 50–20%; - —no inhibition of bacterial culture growth.

## Data Availability

The genome sequence was deposited in GenBank under the accession number OK428535. The raw data supporting the conclusions of this article will be made available by the authors, without undue reservation.

## Supplementary Materials

The following supporting information can be downloaded at: https://www.mdpi.com/article/10.3390/microorganisms11061524/s1, Table S1: Presence of virulent genes in bacterial isolates; Table S2: Antibiotic susceptibility of bacterial isolates.
