# Supplementary material for: Isolation and Characterization of Jumbo Coliphage vB_EcoM_Lh1B as a Promising Therapeutic Agent against Chicken Colibacillosis
Source: Microorganisms. 2023 Jun 8;11(6):1524. doi: 10.3390/microorganisms11061524 (PMC10302289; doi:10.3390/microorganisms11061524)
Supplement: Supplementary file 1 [file microorganisms-11-01524-s001.zip › Supplementary Table S1.pdf]

Table S1 - Presence of virulence genes in bacterial isolates

Isolate ID		Isolation source	virulence factors detected				
			iroN	ompT	hlyF	iss	iutA
E. coli F/18	Avian Pathogenic *E.coli*	various organs of broiler chickens with colibacillosis	+	+	+	+	+
Sor. lv. bc2			+	+	+	+	+
Yun 3d pas			+	+	+	+	+
Sor. lb. sc2			+	+	+	+	+
Int. m/l 04.06			+	+	+	+	+
Sor.br. sc2			+	+	+	+	+
Sor. int. bc2			-	+	+	+	+
Kd. m/l 04.06			+	+	+	+	+
Sor.st. sc2			+	+	+	+	+
Faec.H.bl. Uz.			+	+	+	+	+
Sor.br.bc1			+	+	+	+	+
Sor. kd. sc2			+	+	+	+	+
Sor. lv. sc2			+	+	+	+	+
Sor. br. sc1			+	+	+	+	+
F.H.pr. Uz.			-	+	+	+	+
Int WP #4			+	+	+	+	+
Br. m/l 10.06			+	+	+	+	+
Sor. st. Sc1			-	-	-	+	+
Alag 1 st.			+	+	+	+	+
Sor.st. bc1			-	-	-	+	-
Int WR #4			+	+	+	+	+
Yun#2			+	+	+	+	+
St.m/l 04.06			+	+	+	+	+
Lv. m/l 04.06			+	+	+	+	+
St.m/l 10.06			+	+	+	+	+
F.ch. m/l Uz.			+	+	+	+	+
Sor. lv. Bc1			-	-	-	+	-
Sor. lv. Sc1			+	+	+	+	+
Sor. kd. bc1			-	-	-	-	+
Sor.br.bc2			-	+	+	+	+

PCR method for detecting the virulence genes

Amplification of the virulence genes was carried out following a method described by Johnson *et al* (2008). Briefly, the reaction mixture consisted of; 12,5µL of PCR Master Mix 2X (Thermo Scientific), 0.5µL of each primer (Applied Biosystems), 2 µL of DNA template the total volume of the reaction mixture was adjusted to 20 µl with nuclease-free water. The PCR reactions were performed under the following parameters; 94°C for 2 min; 30 cycles of 94°C for 30 s, 63°C for 30 s, 68°C for 3 min; and a final cycle of 72°C for 10 min. The PCR products were analyzed by horizontal gel electrophoresis in 1% agarose. Agarose gel was stained with SYBR™ Safe DNA Gel Stain (Invitrogen). The primer sequence for the selected virulence genes are presented in the table below.

Primer sequences and the amplicon length of the *E. coli* virulence genes

Gene	Primer Sequence	Amplicon length (bp)
iroN	F: AATCCGGCAAAGAGACGAACCGCCT	553

Salmochelin siderophore receptor gene	R: GTTCGGGCAACCCCTGCTTTGACTTT	
ompT<br>*Episomal outer membrane protease gene*	F: TCATCCCGGAAGCCTCCCTCACTACTAT<br>R: TAGCGTTTGCTGCACTGGCTTCTGATAC	496
hlyF<br>*Putative avian hemolysin*	F: GGCCACAGTCGTTTAGGGTGCTTACC<br>R: GGCGGTTTAGGCATTCCGATACTCAG	450
Iss<br>*Episomal increased serum survival gene*	F: CAGCAACCCGAACCACTTGATG<br>R: AGCATTGCCAGAGCGGCAGAA	323
iutA<br>*Aerobactin siderophore receptor gene*	F: GGCTGGACATCATGGGAACTGG<br>R: CGTCGGGAACGGGTAGAATCG	302
