# Supplementary material for: Isolation and Characterization of Jumbo Coliphage vB_EcoM_Lh1B as a Promising Therapeutic Agent against Chicken Colibacillosis
Source: Microorganisms. 2023 Jun 8;11(6):1524. doi: 10.3390/microorganisms11061524 (PMC10302289; doi:10.3390/microorganisms11061524)
Supplement: Supplementary file 1 [file microorganisms-11-01524-s001.zip › Supplementary Table S2.pdf]

Table S2 - Antibiotic Susceptibility of Bacterial Isolates

Isolate ID	Antibiotics							
	PIT	MER	CEP	CAZ	CZC	AZT	TGC	NET
E. coli F/18	S	S	R	R	S	R	S	S
Sor. lv. bc2	S	S	S	S	S	R	S	S
Yun 3d pas	S	S	S	S	S	R	S	S
Sor. lb. sc2	S	S	S	S	S	R	S	S
Int. m/l 04.06	S	S	S	S	S	R	S	S
Sor.br. sc2	S	S	S	S	S	I	S	S
Sor. int. bc2	S	S	S	S	S	R	S	S
Kd. m/l 04.06	S	S	S	S	S	R	S	S
Sor.st. sc2	S	S	S	R	R	R	S	S
Faec.H.bl. Uz.	S	S	S	S	S	R	S	S
Sor.br.bc1	S	S	S	R	R	R	S	S
Sor. kd. sc2	S	S	S	S	S	R	S	S
Sor. lv. sc2	S	S	S	S	S	R	S	S
Sor. br. sc1	S	S	S	R	R	R	S	S
F.H.pr. Uz.	S	S	S	S	S	R	S	S
Int WP #4	R	S	R	R	R	R	S	R
Br. m/l 10.06	S	S	S	I	R	R	S	S
Sor. st. Sc1	S	S	S	I	R	R	S	S
Alag 1 st.	S	S	S	I	R	R	S	S
Sor.st. bc1	S	S	S	S	S	R	S	S
Int WR #4	S	S	S	S	I	S	S	S
Yun#2	S	S	S	S	S	R	S	S
St.m/l 04.06	S	S	S	R	R	R	S	I
Lv. m/l 04.06	R	S	S	R	R	R	S	I
St.m/l 10.06	S	S	S	R	R	R	S	S
F.ch. m/l Uz.	S	S	S	S	S	R	S	S
Sor. lv. Bc1	R	R	R	I	S	R	S	S
Sor. lv. Sc1	S	S	S	I	I	R	S	S
Sor. kd. bc1	S	S	S	R	R	R	S	S
Sor.br.bc2	S	S	S	I	R	R	S	S

S – sensitive; R – resistant; I - intermediate

Ureidopenicillins (PIT - piperacillin/tazobactam)
Carbapenems (MER – meropenem
4th generation cephalosporins (CEP – cefepime)
3rd generation cephalosporins (CAZ – ceftazidime , CZC - ceftazidime / clavulanate)
Monobactam group (AZT – aztreonam)
Glicylcyclines (TGC – tigecycline)
Aminoglycosides (NET – netilmicin)

The technique included rehydrating wells with antibiotics using a suspension medium and introducing a bacterial suspension (0.5 MFU density) into them. The results of the sensitivity of microorganisms were taken into account after 18-24 hours of incubation visually or photometrically by the presence of bacterial growth in the microwells of the plate.
